# Function and clinical significance of circRNAs in solid tumors

**DOI:** 10.1186/s13045-018-0643-z

**Published:** 2018-07-31

**Authors:** Yiting Geng, Jingting Jiang, Changping Wu

**Affiliations:** 1 Department of Oncology, The Third Affiliated Hospital of Soochow University, 185 Juqian Street, Changzhou, 213003 Jiangsu China; 2 Department of Tumor Biological Treatment, The Third Affiliated Hospital of Soochow University, 185 Juqian Street, Changzhou, 213003 Jiangsu China

**Keywords:** circRNA, microRNA sponge, Solid tumors, Biomarker

## Abstract

Circular RNA (circRNA) is a new type of endogenous non-coding RNAs (ncRNAs). circRNA regulates gene expression in many biological processes, and it also participates in the initiation and development of various diseases, including tumors, which are the focus of present research. With the development of high-throughput sequencing technique, an increasing number of circRNAs closely related to tumors have been discovered. According to numerous studies, there is a significant difference in the expressions of circRNAs among a variety of tumor tissues and para-carcinoma normal tissues. Some specifically expressed circRNAs may potentially serve as new biomarkers for tumor diagnosis and prognosis. This systemic review briefly introduces the characteristics, biogenesis, and functions of circRNAs, as well as discusses their relationship with cancer in detail. In addition, this article also describes several research strategies for circRNAs.

## Background

More than 70% human genomes are transcribed, and protein-coding genes only account for 1–2%. Most transcripts are non-coding RNAs (ncRNAs) [1]. Circular RNA (circRNA) is a type of new ncRNA different from linear RNA as it is a continuous covalently closed loop without the 5′-cap structure and the 3′-poly A tail. Most circRNAs are universal, stable, and conserved, and they are often specifically expressed in different tissues and developmental stages. In 1979, Hsu and Coca-Prados at Rockefeller University observed that circRNA exists in the cytoplasm of eukaryotic cells [2]. Within decades after the 1970s, circRNA has been considered as an outcome of a splicing error. With the development of RNA sequencing (RNA-seq) technology and bioinformatics in the twenty-first century, a large number of circRNAs have been discovered. There are four types of circRNAs, namely, exonic circRNA (ecircRNA), circRNAs from introns, exon-intron circRNA (EIciRNA), and intergenic circRNA [3]. More than 80% of the circRNAs are ecircRNAs, which are formed by the reverse covalent attachment of the 3′ splice donors and the 5′ splice acceptors of the precursor mRNA (pre-mRNA). circRNAs from introns are a general term for a class of circRNAs, including circular intronic RNAs (ciRNAs), excised group I introns, excised group II introns, excised tRNA introns, and intron lariats. EIciRNA is a type of circRNAs that are circularized simultaneously by exons and introns, probably similar to ecircRNAs. Intergenic circRNA is another non-exonic circRNA found by circRNA Identifier (CIRI). This integrated circRNA is formed by two intronic circRNA fragments (ICFs) flanked by GT-AG splicing signals acting as the splice donor (SD) and splice acceptor (SA) of the circular junction.

**Fig. 1:**
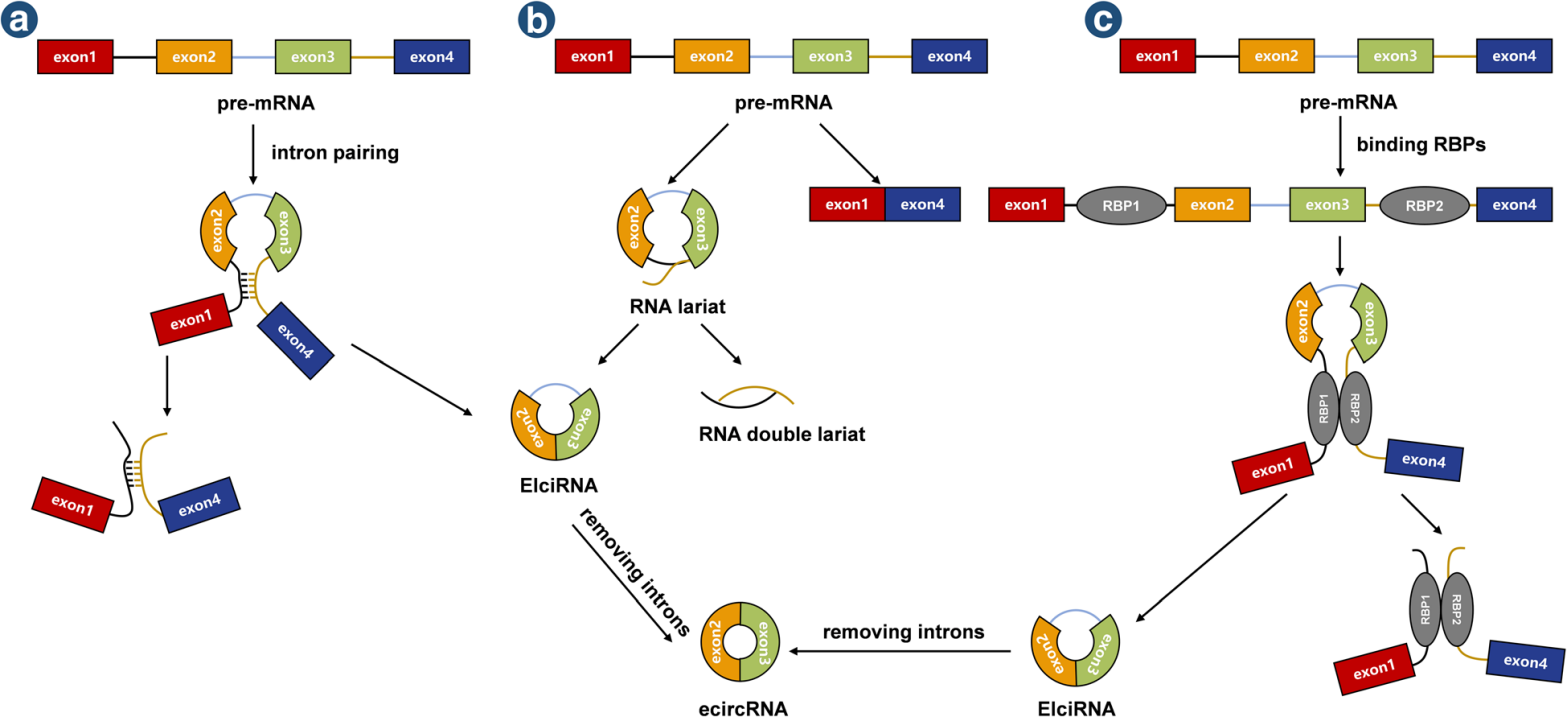
Biogenesis of ecircRNAs and EIciRNAs. **a** Intron-pairing-driven circularization: The introns flanking inverted repeats or ALU elements form a circular RNA by base pairing, and ecircRNAs or EIciRNAs are formed by removing or retaining introns. **b** Lariat-driven circularization: EcircRNAs or EIciRNAs are generated by exon skipping. Exons 5′ attacks the 3′ splice site, forming an RNA lariat containing skipped exons 2 and 3 and an mRNA consisting of exon 1 and exon 4. Then, an EIciRNA and an RNA double lariat were further formed. **c** circRNA biogenesis depends on RBPs: RBPs bind to the sequence motifs of upstream and downstream introns, which serve as a bridge to bring flanking introns closer to each other, promoting the formation of circRNAs

**Fig. 2:**
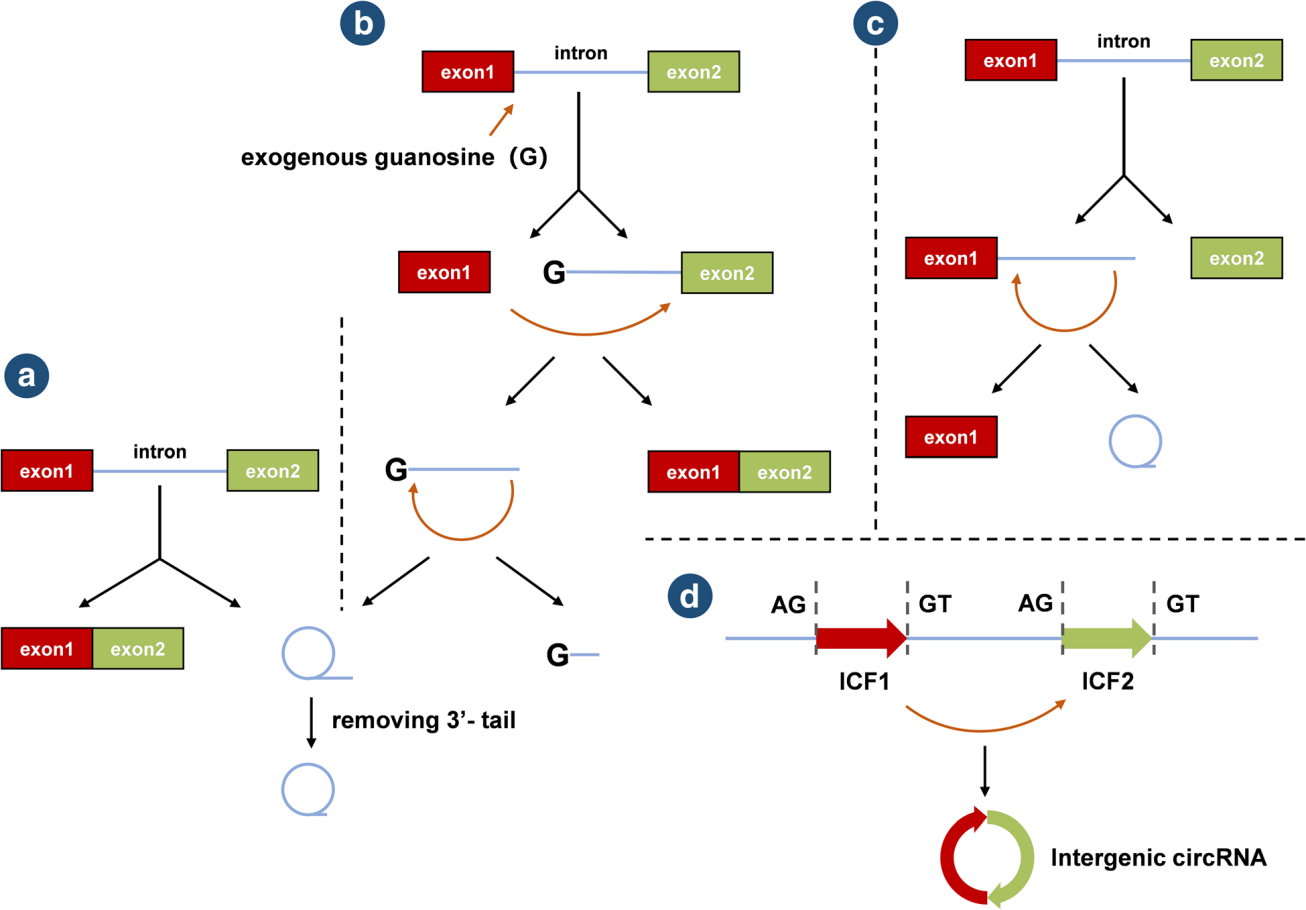
Biogenesis of circRNAs from introns and intergenic circRNAs. **a** ciRNA: The pre-mRNA is spliced to produce an RNA lariat with a 2′,5′-phosphodiester, and the ciRNA is formed upon removal of the 3′-tail. **b** Excised group I introns: Exogenous guanosine (G) attacks the 5′-terminus of the intron, and exon 1 is cleaved due to transesterification. Then, the 5′-terminus of exon 2 is attacked by the 3′-hydroxyl of free exon 1, creating a linear intron. The 2′-hydroxyl close to the 3′-terminus of this linear intron attacks the phosphodiester bond near the 5′-terminus, resulting in an RNA lariat with 2′,5′-phosphodiester and a released 5′-terminus sequence. After removing the 3′-tail of the RNA lariat, a circRNA from group I introns is formed. **c** Excised group II introns: Pre-mRNA releases exon 2, and the 2′-hydroxyl of the intron’s 3′-terminus attacks the phosphodiester bond near the 5′-terminus, producing an RNA lariat with 2′,5′-phosphodiester and the free exon 1. **d** The circRNA contains two ICFs flanked by GT-AG splicing signals which act as SD and SA of the circular junction to form an integrated circRNA

Two basic models of circRNA biogenesis have been proposed as follows: (1) intron-pairing-driven circularization, also known as direct back-splicing (Fig. 1a), is the main form of ecircRNA production, in which the flanking intronic complementary sequences of the pre-mRNA form a lariat by direct base-pairing, forming an ecircRNA when introns are removed, and (2) lariat-driven circularization, also known as exon-skipping (Fig. 1b), in which the pre-mRNA is partially folded during transcription, allowing the 3′-SD of the downstream exon to connect to the 5′-SA of the upstream exon, resulting in exon-skipping and the formation of a RNA lariat containing both exons and introns. With the removal of introns, an ecircRNA is formed. Generally, introns between circular exons will be excised. However, in some cases, these introns are retained as EIciRNAs. Some introns containing key nucleotide sequences are not decomposed by the debranching enzyme after splicing but instead independently cyclize into ciRNAs, which is called intron cyclization. In addition, there is another pattern of circRNA biogenesis that depends on RNA-binding proteins (RBPs) (Fig. 1c). The formation of circRNAs from introns and intergenic circRNAs is detailed in Fig. 2a-c and d, respectively.

circRNA has several features as follows: (1) abundance and diversity: more than 20,000 different circRNAs have been identified in eukaryotes; (2) stability: the half-life of circRNA is long because circRNA is a covalently closed circular structure without a 5′-cap and 3′-poly A tail, which is not easily degraded by exonuclease, resulting in far superior stability of circRNA than that of linear mRNA; (3) conservation: circRNA is highly conserved among different species, such as humans, mice, nematodes, zebrafish, drosophila, protists, and plants;(4) positioning: ecircRNA accounts for the majority of all circRNA types, which mainly exist in the cytoplasm, and intron-containing circRNAs including ciRNA and EIciRNA, which are mainly located in the nuclei of eukaryotes; and (5) specificity: circRNA is often specifically expressed in different tissues and different developmental stages.

**Fig. 3:**
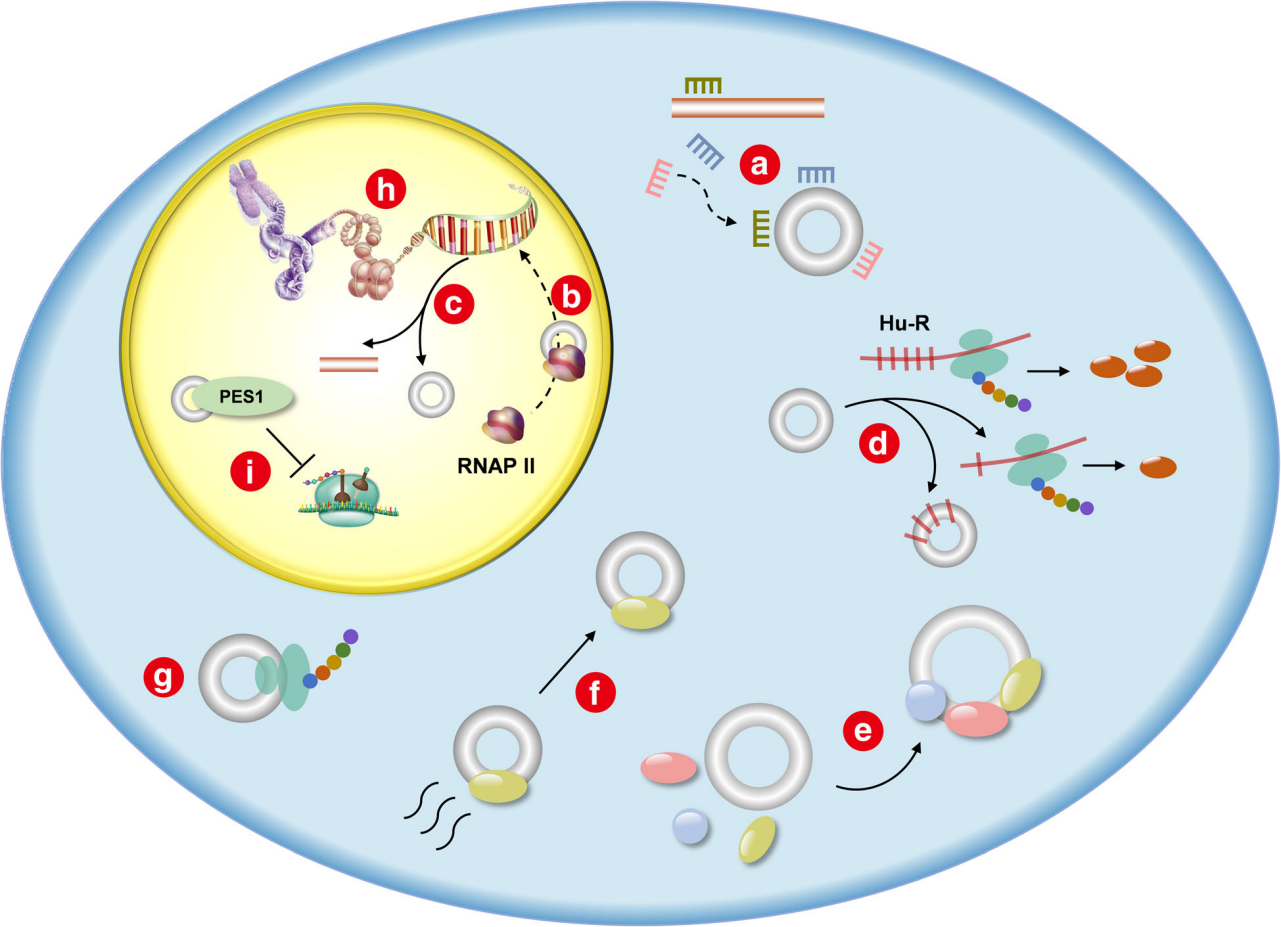
Functions of circRNAs: **a** miRNA sponge. **b** RNAP II elongation. **c** Alternative splicing. **d** Translation regulation. **e** Protein scaffold. **f** Protein localization. **g** Translation template. **h** Histone modification. **i** RNA maturation

circRNA has abundant biological functions and is involved in various physiological and pathological processes of tumor cells, including proliferation, apoptosis, invasion, and migration. One of the most frequently studied functions of circRNA is the microRNA (miRNA) sponge [4–6], namely, eliminating the miRNA’s regulation of a target gene via binding to the miRNA as a competing endogenous RNA (ceRNA) through the base complementary pairing principle. Moreover, circRNAs can also regulate gene expression at the transcriptional and post-transcriptional levels through other mechanisms, and they play a role as RBP sponges and protein scaffolds [7], some of which even have the ability to translate proteins [8, 9]. In addition, circRNAs are involved in RNAP II elongation [10, 11], alternative splicing [12], translation regulation [13], protein localization [14], histone modification [15, 16], and RNA maturation [17]. Recent studies have shown that circRNAs exert their biological functions through various mechanisms (Table 1, Fig. 3).

**Table 1:** Functions of circRNAs

Function	Figure 1	Representative circRNA	Reference
miRNA sponge	a	circHIPK3	[4]
		ciRS-7	[5, 6]
RNAP II elongation	b	ci-ankrd52	[10]
		EIciEIF3j	[11]
Alternative splicing	c	circMbl	[12]
Translation regulation	d	circPABPN1	[13]
Protein scaffold	e	circ-Foxo3	[7]
Protein localization	f	circ-Foxo3	[14]
Translation template	g	circ-ZNF609	[8]
		circMbl	[9]
Histone modification	h	cANRIL	[15, 16]
RNA maturation	i	circANRIL	[17]

More and more circRNAs have been reported to be dysregulated in many human malignancies, such as lung cancer, breast cancer, gastric cancer, colorectal cancer, and liver cancer; they may serve as new diagnostic biomarkers and targets for cancer therapy. In this paper, we performed a systematic review of literature to provide information about the expression patterns and roles of circRNAs in solid tumors.

## Main text

One hundred eighteen articles that investigated the expressions of circRNAs in various solid tumors were selected for the systemic review through searching PubMed, Embase, and Cochrane libraries (as of March 26, 2018), including nine cases of lung cancer, 14 cases of breast cancer, three cases of esophageal squamous cell carcinoma (ESCC), 20 cases of gastric cancer, 17 cases of colorectal carcinoma (CRC), 16 cases of hepatocellular carcinoma (HCC), 10 cases of gliomas, seven cases of bladder cancer, four cases of pancreatic cancer, five cases of osteosarcomas, and 14 cases of other tumors [two cases of ovarian cancer, one case of kidney cancer, one case of thyroid cancer, one case of basal cell carcinoma (BCC), one case of cutaneous squamous cell carcinoma (CSCC), two cases of oral squamous cell carcinoma (OSCC), one case of laryngeal squamous cell cancer (LSCC), one case of hypopharyngeal cancer, two cases of cholangiocarcinoma, one case of cervical cancer, and one case of prostate cancer].

### Lung cancer

Zhao et al. screened four pairs of high-throughput circRNA microarrays of lung cancer and para-carcinoma tissues and found that 357 circRNAs are dysregulated [18]. circ-ITCH, a sponge of many oncogenic miRNAs, plays an important inhibitory role in the progression of lung cancer. Wan et al. detected the circ-ITCH levels in cancer tissues and para-carcinoma tissues in 78 lung cancer cases, and they reported that circ-ITCH expression is significantly reduced in approximately 73% of lung cancer tissues. Overexpression of circ-ITCH inhibits the proliferation of lung cancer cells and is associated with the expressions of host genes [19]. Another non-small cell lung cancer (NSCLC) study showed that the expression of circRNA_100876 (circ-CER) is significantly upregulated in cancer tissues. Such high expression of circ-CER is significantly associated with local lymph node invasion and advanced tumor. Patients with high circ-CER expression have significantly worse overall survival (OS) than those with low circ-CER expression [20]. Functional experiments have shown that circ-CER may be involved in the growth, progression, and metastasis of NSCLC cells [21]. Therefore, circ-CER may serve as a good diagnostic marker of NSCLC, and it is also a potential therapeutic target. In addition, hsa_circ_0013958 from the ACP6 gene is overexpressed in lung adenocarcinoma and correlated with tumor TNM staging [22]. Hsa_circ_0012673 is also upregulated in lung adenocarcinoma tissues and mainly located in the cytoplasm, regulating the proliferation of lung adenocarcinoma cells by adsorbing miR-22 [23]. Hsa_circ_0007385 is overexpressed in both NSCLC tissues and cells. Downregulation of hsa_circ_0007385 significantly inhibits the proliferation and invasion of NSCLC [24]. Hsa_circ_0014130 is highly expressed in NSCLC tissues and is closely related to lymph node metastasis and TNM staging, which can be used for prognostic evaluation [25]. In addition, the overexpression of hsa_circ_0000064 in lung cancer is positively correlated with T and N stage. Knockout of hsa_circ_0000064 significantly inhibits cell proliferation and promotes apoptosis [26]. The expression and function of circRNAs in lung cancer are shown in Table 2.

**Table 2:** circRNAs in lung cancer

circBase ID (alias)	Host gene	Putative function	Upregulated/downregulated	miRNA sponge	Target gene/pathway	Reference
hsa_circ_0013958	ACP6	miRNA sponge	Upregulated	miR-134	–	[22]
circ-ITCH	ITCH	miRNA sponge	Downregulated	miR-7, miR-214	Wnt/β-Catenin	[19]
circ-CER	CER	miRNA sponge	Upregulated	miR-136	MMP13	[20]
						[21]
hsa_circ_0007385	MEMO1	miRNA sponge	Upregulated	miR-181	–	[24]
hsa_circ_0012673	DHCR24	miRNA sponge	Upregulated	miR-22	ErbB3	[23]
hsa_circ_0014130	PIP5K1A	miRNA sponge*	Upregulated	–	–	[25]
hsa_circ_0000064	B4GALT2	miRNA sponge*	Upregulated	–	MMP-2, MMP-9	[26]

*Not validated experimentally

### Breast cancer

Approximately 20% of breast cancers detected by mammography are ductal carcinomas in situ (DCIS) [27]. Some of these highly curative tumors will develop into invasive ductal carcinoma (IDC), which is life-threatening. However, the underlying determinants still remain unclear. A recent study has identified two circRNAs (hsa_circ_0122662 and hsa_circ_0001358) in five patients with DCIS/IDC and the MCF-7 invasive breast cancer cell line. Five miRNAs (miR-200b-3p, miR-200c-3p, miR-376a-3p, miR-376b-3p, and miR-429) have been confirmed to bind to hsa-circ-0001358 [28]. Further study of differentially expressed circRNAs in DCIS/IDC can aid the understanding of the molecular mechanisms underlying the progression from DCIS to IDC.

Nair et al. found 411 tumor-specific circRNAs from 885 breast cancer samples from TCGA in triple-negative (TN) breast cancer, including 256 estrogen receptor-positive (ER+), and 288 HER-2-positive (HER-2+) breast cancer cases [29]. Lu et al. found that 715 out of 1155 differentially expressed circRNAs are upregulated in breast cancer tissues compared to para-carcinoma tissues but that the other 440 circRNAs are downregulated. Validation studies have shown that hsa_circ_103110, hsa_circ_104821, and hsa_circ_104689 are upregulated in breast cancer tissues but that hsa_circ_100219, hsa_circ_006054, and hsa_circ_406697 are downregulated. The combination of hsa_circ_006054, hsa_circ_100219, and hsa_circ_406697 provides valuable insights into the diagnosis of breast cancer [30]. Some scholars believe higher diagnostic value of circRNAs in breast cancer than CEA and CA-153 [31].

circ-Foxo3 is a potential tumor suppressor that is significantly downregulated in breast cancer tissues [32] and may be involved in tumor progression [33]. Overexpression of circ-Foxo3 in the MDA-MB-231 breast cancer cell line significantly reduces cell proliferation in vitro. Subcutaneous injection of MDA-MB-231 cells transfected with circ-Foxo3 into nude mice inhibits tumor growth and promotes apoptosis [32]. A total of 25 binding sites of circ-Foxo3 for eight miRNAs (miR-22, miR-136, miR-138, miR-149, miR-433, miR-762, miR-3614-5p, and miR-3622b-5p) are detected [34], and transfection of these miRNAs into MDA-MB-231 cells can reduce apoptosis.

circ-VRK1 is also one of the downregulated circRNAs in breast cancer, especially in breast cancer stem cells (BCSCs). Upregulation of circ-VRK1 will inhibit the stemness of BCSCs [35]. In addition, circ_000911 is poorly expressed in breast cancer. In vitro experiments have confirmed that upregulation of circ_000911 increases Notch1 expression via binding to miR-449a, thereby suppressing the proliferation, invasion, and metastasis of breast cancer cells [36].

In contrast, hsa_circ_0001982, hsa_circ_0005239, and hsa_circ_0008717 are upregulated in breast cancer, and knockdown of their expressions inhibits cell proliferation and promotes apoptosis [37–39]. circ_0006528 is highly expressed in chemotherapy-resistant breast cancer cell lines, and the sensitivity of these cells to chemotherapy is significantly increased after knocking down circ_0006528 [40]. The expression of circ-DENND4C is increased in breast cancer cell lines under hypoxic conditions, and downregulation of circ-DENND4C inhibits the proliferation of breast cancer cells [41]. The expression and function of circRNAs in breast cancer are shown in Table 3.

**Table 3:** circRNAs in breast cancer

circBase ID (alias)	Host gene	Putative function	Upregulated/downregulated	miRNA sponge	Target gene/pathway	Reference
circ-Foxo3	FOXO3	Protein scaffolding	Downregulated	–	p53	[32]
circ-Foxo3	FOXO3	miRNA sponge	Downregulated	miR-22, miR-136, miR-138, miR-149, miR-433, miR-762, miR-3614-5p, miR-3622b-5p	–	[34]
hsa_circ_0008717	ABCB10	miRNA sponge	Upregulated	miR-1271	–	[37]
hsa_circ_0001358	SEC62	miRNA sponge*	Upregulated	–	ZEB1/2	[28]
hsa_circ_000911	IFNGR2	miRNA sponge	Downregulated	miR-449a	Notch1	[36]
hsa_circ_0001982	RNF111	miRNA sponge	Upregulated	miR-143	–	[38]
hsa_circ_0005239	GFRA1	miRNA sponge	Upregulated	miR-34a	GFRA1	[39]
hsa_circ_0006528	PRELID2	miRNA sponge	Upregulated	miR-7	Raf1	[40]
circ-DENND4C	DENND4C	–	Upregulated	–	HIF1α	[41]

*Not validated experimentally

### ESCC

Several dysregulated circRNAs are found in ESCC, including hsa_circ_000167, hsa_circ_001059, hsa_circ_0067934, and circ-ITCH [42–44]. Similar to lung cancer, downregulation of circ-ITCH is also observed in 684 ESCC tissues and para-carcinoma tissues [42]. circRNAs may be associated with the radio-resistance of ESCC. In a circRNA microarray analysis of radiation-sensitive and radio-resistant cells, researchers have found that 57 significantly upregulated circRNAs and 17 downregulated circRNAs in radio-resistant ESCC cells, excluding circ-ITCH. KEGG analysis has shown that more than 400 differentially expressed target genes of circRNAs are enriched in the Wnt signaling pathway. Su et al. identified more than 3700 human circRNAs, among which hsa_circ_000167 and hsa_circ_001059 in the KYSE-150R human radiation-resistant esophageal cancer cell line are significantly different from the KYSE-150 parental cell line. These two circRNAs were confirmed by circRNA-miRNA co-expression analysis to be the most important factors in the potential circRNA/miRNA networks [44]. Xia et al. found that hsa_circ_0067934 encoded by PRKCI is upregulated in 51 cases of ESCC tissues compared to adjacent noncancerous tissues, and they reported that hsa_circ_0067934 is associated with poor tumor differentiation and advanced TNM stage. Silencing hsa_circ_0067934 by siRNA induces cell cycle arrest and inhibits proliferation and migration of ESCC cells [43]. Given that TNM staging is applied to predict patient outcomes, hsa_circ_0067934 may serve as a potential prognostic marker for ESCC. The expression and function of circRNAs in ESCC are shown in Table 4.

**Table 4:** circRNAs in ESCC

circBase ID (alias)	Host gene	Putative function	Upregulated/downregulated	miRNA sponge	Target gene/pathway	Reference
hsa_circ_0067934	PRKCI	–	Upregulated	–	–	[43]
hsa_circ_0000554	PRB4	miRNA sponge*	Downregulated	miR-30c-1, miR-30c-2, miR-122, miR-139-3p, miR-339-5p, miR-1912	–	[44]
hsa_circ_0000518	RPPH1	miRNA sponge*	Downregulated	miR-181a-2, miR-512-5p, miR-521, miR-556-5p, miR-663b, miR-1204	–	[44]
circ-ITCH	ITCH	miRNA sponge		miR-7, miR-17, miR-214	Wnt/β-catenin	[42]

*Not validated experimentally

### Gastric cancer

Several studies have examined the differential expression of circRNAs between gastric cancer and para-carcinoma tissues by circRNA microarrays [45–50]. Chen et al. found 180 circRNAs differentially expressed in gastric cancer and normal tissues using RNA-seq analysis. Among which, circ-PVT1 is upregulated, and the overexpression of circ-PVT1 suggests a better OS and disease-free survival (DFS). A luciferase reporter assay has confirmed that circ-PVT1 indirectly regulates the expression of the E2F2 transcription factor as a sponge of miR-125 family, promotes the colony formation, and is involved in cell cycle regulation [45].

Hsa_circ_0047905, hsa_circ_0087198, and hsa_circ_0138960 are also highly expressed in gastric cancer tissues. Inhibition of these circRNAs significantly suppresses the proliferation of gastric cancer cells [50]. Sui et al. found that hsa_circRNA_000792 and hsa_circRNA_400071 are upregulated in gastric cancer but that hsa_circRNA_001066, hsa_circRNA_001959, and hsa_circRNA_400066 are downregulated [49].

Hsa_circ_0000096, also known as circ-HIAT1, is downregulated in gastric cancer cells and tissues. Knockdown of hsa_circ_0000096 reduces the expression of cyclin D1, CDK6, matrix metalloproteinase (MPP)-2, and MMP-9, and it significantly inhibits cell proliferation and migration and blocks cell cycle (preventing gastric cancer cells from leaving G0/G1 phase to enter S phase), as well as inhibits tumor growth in a xenograft nude mouse model. The circRNA database shows that hsa_circ_0000096 can interact with 17 different types of miRNAs. Downregulation of hsa_circ_0000096 results in a decrease in miR-224 (a modulator of CD40) and an increase in miR-200a (targeting E-cadherin) [51].

Both circ-LPHN2 and circ-LARP4 are lowly expressed in gastric cancer tissues. The former acts as a sponge of miR-630 and inhibits the proliferation of gastric cancer cells [52]. The latter exerts biological functions by adsorbing miR-424, and it acts as an independent prognostic factor for gastric cancer [53]. Hsa_circ_002059 has also been confirmed to be downregulated in gastric cancer tissues. The low expression of hsa_circ_002059 is significantly associated with gender, age, distant metastasis, and TNM staging [54]. In particular, the postoperative level of plasma hsa_circ_002059 in gastric cancer patients is lower than that before surgery. Hsa_circ_0000190 is downregulated in gastric cancer tissues and plasma, and its low expression level is related to tumor size, lymph node and distant metastasis, and TNM staging. Hsa_circ_0000190 is considered to have better sensitivity and specificity compared to CEA and CA19-9 [55]. The low expression of hsa_circ_0001895 in gastric cancer tissues is significantly associated with histological types and grade [56]. Hsa_circ_0014717 is also lowly expressed in gastric cancer, and such downregulation is associated with distant metastasis and clinical staging. Additionally, hsa_circ_0014717 can be stably detected in gastric juice [48]. Hsa_circ_0000181, hsa_circ_0001649, hsa_circ_0000520, hsa_circ_0003159, and hsa_circ_0074362 are also lowly expressed in tissues or plasma of gastric cancer patients, and their expression is negatively correlated with distant metastasis and TNM staging. Therefore, these circRNAs may be used as diagnostic indexes to indicate if there is distant metastasis [57–61]. The plasma levels of hsa_circ_0001017 and hsa_circ_0061276 are also downregulated, making them suitable for the diagnosis and prognosis of gastric cancer [62]. Moreover, hsa_circ_0000745 is expressed at a higher level in gastric cancer tissues than normal tissues, and its expression in plasma of gastric cancer patients is also higher than that of healthy controls. Hsa_circ_0000745 expression in gastric cancer tissues and plasma is associated with tumor differentiation and lymph node metastasis, respectively, and plasma hsa_circ_0000745 combined with CEA has a greater diagnostic value for gastric cancer [63]. The expression of circRNA may be a predictor of early recurrence in patients with radical resection of stage III gastric cancer [64], but the major shortcoming of this previous study was that the follow-up time was too short. The expression and function of circRNAs in gastric cancer are shown in Table 5.

**Table 5:** circRNAs in gastric cancer

circBase ID (alias)	Host gene	Putative function	Upregulated/downregulated	miRNA sponge	Target gene/pathway	Reference
hsa_circ_0001821	PVT1	miRNA sponge	Upregulated	miR-125a/b	–	[45]
hsa_circ_0000190	CNIH4	–	Downregulated	–	–	[55]
hsa_circ_0000096	HIAT1	–	Downregulated	–	CDK6, MMP9, MMP2	[51]
hsa_circ_002059	KIAA0907	–	Downregulated	–	–	[54]
hsa_circ_0014717	CCT3	–	Downregulated	–	–	[48]
hsa_circ_0001895	PRRC2B	–	Downregulated	–	–	[56]
hsa_circ_0003159	CACNA2D1	–	Downregulated	–	–	
hsa_circ_0000520	RPPH1	miRNA sponge*	Downregulated	–	–	[59]
hsa_circ_0001649	SHPRH	–	Downregulated	–	–	[57]
hsa_circ_0074362	ARHGAP26	–	Downregulated	–	–	[58]
hsa_circ_0061276	NRIP1	–	Downregulated	–	–	[62]
hsa_circ_0001017	XPO1	–	Downregulated	–	–	[62]
hsa_circ_0000181	TATDN3	–	Downregulated	–	–	[60]
hsa_circ_0000745	SPECC1	–	Downregulated	–	–	[63]
hsa_circ_101057	LARP4	miRNA sponge	Downregulated	miR-424	LATS1	[53]
circ-LPHN2	LPHN2	miRNA sponge	Downregulated	miR-630	–	[52]
hsa_circ_0014717	CCT3	–	Downregulated	–	–	[48]
hsa_circ_0047905	SERPINB5	–	Upregulated	–	–	[50]
hsa_circ_0138960	GDA	–	Upregulated	–	–	[50]
hsa_circ_0087198	GDA	–	Upregulated	–	–	[50]

*Not validated experimentally

### CRC

Bachmayr-Heyda et al. found that 11 circRNAs are upregulated and 28 circRNAs are downregulated in CRC tissues compared to para-carcinoma tissues by RNA-seq analysis [65]. In addition, the ratio of some circRNAs (circ3204/USP3, circ0817/CUL5, circ7374/TNS4, and circ6229/METTL3) to linear RNA is lower in CRC tissues than in normal tissues. Zhang et al. [66] also found that there are more circRNAs downregulated in CRC tissues and that the most significant downregulated circRNA is derived from the PTK2 tumor suppressor gene. This phenomenon can be attributed to the high stability of circRNA as it accumulates in non-proliferating cells and is dispersed in daughter cells of proliferating cells [65].

Similar to lung cancer, circ-ITCH is also significantly downregulated in CRC tissues. circ-ITCH is a sponge of some miRNAs which downregulate many target genes involved in G1/S transition, including miR-7, miR-20a, and miR-214. Overexpression of circ-ITCH reduces the proliferation of SW480 and HCT116 cells. Therefore, circ-ITCH may have anti-proliferative effects in CRC [67].

The downregulation of Hsa_circ_001988 in CRC is significantly related to differentiation and neural invasion of cancer cells. Because nerve invasion is a definite negative prognostic factor in CRC patients, hsa_circ_001988 may be a promising prognostic biomarker of patients with CRC [68]. In addition, hsa_circ_0001649, hsa_circ_0003906, and circRNA derived from ITCH78 are also downregulated in CRC, and the first two circRNAs are related to the pathological differentiation of CRC and may be used as diagnostic indicators of CRC [69, 70].

In contrast, the expression of ciRS-7 is upregulated in CRC, and it is the most significantly upregulated circRNA, deriving from METTL3, a m6A methyltransferase gene [65]. A large study by Weng et al., including 153 trial cohorts and 165 validation cohorts, has also confirmed the upregulation of ciRS-7 in CRC tissues. The high expression of ciRS-7 is positively correlated with tumor size, lymph node metastasis, TNM staging, and OS of patients [71]. Knockdown of ciRS-7 inhibits the activity of miR-7 target genes, such as EGFR and IGF-1R, thereby suppressing the proliferation and invasion of CRC.

As a positive regulator of CRC cell proliferation and invasion, hsa_circ_001569 exhibits higher expression in CRC tissues than noncancerous tissues [65, 72, 73]. Hsa_circ_001569, the sponge of miR-145, increases the number of cells in S and G2/M phase and accelerates the proliferation and invasion of CRC cells by preventing miR-145 from downregulating target genes, such as E2F5, FMNL2, and BAG4 [72]. Knockdown of hsa_circ_001569 in LOVO and SW620 cells reverses the invasive ability [72].

Hsa_circ_0000069 is also overexpressed in CRC tissues. siRNA-mediated knockdown of hsa_circ_0000069 inhibits the proliferation, migration, and invasion of HT-29 cells and induces G0/G1 phase arrest [74]. The expression of circRNAs derived from STIL60 and BANP79 genes in CRC tissues is also higher than that in normal tissues. Knockdown of circ-BANP reduces the proliferation and colony formation of the HCT116 and HT29 cell lines. Moreover, the expression of p-Akt is also decreased, suggesting that the PI3K-Akt pathway may be involved in circ-BANP-induced cell proliferation [75]. Zeng et al. found that circ-HIPK3 is overexpressed in CRC cells and tissues, and they reported that the prognosis of CRC patients with high circ-HIPK3 expression is poor. Knockdown of circ-HIPK3 significantly inhibits the proliferation, invasion, and metastasis of CRC. Additionally, changes in cell function induced by circ-HIPK3 are reversed by miR-7 [76]. The high expression of hsa_circ_0007534 in CRC tissues is associated with lymph node metastasis and tumor staging. Interfering with the expression of hsa_circ_0007534 significantly inhibits the proliferation and promotes the apoptosis of CRC cells [77]. Hsa_circ_000984 is also significantly overexpressed in CRC tissues and cells, and knockdown of hsa_circ_000984 reduces the proliferation, invasion, and metastasis of CRC cells. Moreover, competitively binding to miR-106b as a ceRNA, hsa_circ_000984 effectively upregulates CDK6 expression, thereby affecting the function of CRC cells [78]. Hsa_circ_0020397 is highly expressed in CRC cells, which promotes CRC cell proliferation and invasion. In addition, hsa_circ_000984 upregulates the expression of oncogenic TERT and PD-L1 by binding to miR-106b [79].

Hsiao et al. found that circCDK13, circCCNB, and circCCDC66 are upregulated in CRC tissues. The expression of circ-CCDC66 has been detected in various tumor cell lines. circ-CCDC66 acts as a miRNA sponge to protect MYC mRNA from the miRNA-33b- and miR-93-mediated degradation, and it is involved in cell proliferation, migration, and invasion [80].

Comparison of three syngeneic CRC cell lines with different KRAS mutation status, including DLD-1, DKO-1, and DKs-8, has shown that extracellular circRNAs are more abundant than intracellular circRNAs, and most circRNAs are downregulated in the KRAS-mutated CRC cell lines [81]. circRNA is associated with KRAS mutations, and it is a promising biomarker of CRC, especially for KRAS-mutated CRC. The expression and function of circRNAs in CRC are shown in Table 6.

**Table 6:** circRNAs in CRC

circBase ID (alias)	Host gene	Putative function	Upregulated/downregulated	miRNA sponge	Target gene/pathway	Reference
hsa_circ_0000523	METTL3	–	Downregulated	–	–	[65]
hsa_circ_0001346	RNF13	–	Downregulated	–	–	[65]
hsa_circ_0001793	IKBKB	–	Upregulated	–	–	[65]
hsa_circ_0001946	CDR1	–	Upregulated	–	–	[65]
hsa_circ_0001946	CDR1	miRNA sponge	Upregulated	miR-7	EGFR, RAF1	[71]
hsa_circ_0000069	STIL	–	Upregulated	–	–	[74]
circ-CCDC66	CCDC66	miRNA sponge	Upregulated	miR-33b, miR-93	–	[80]
hsa_circ_001569	ABCC1	miRNA sponge	Upregulated	miR-145	E2F5, BAG4, FMNL2	[72]
circ-ITCH	ITCH	miRNA sponge	Downregulated	miR-7, miR-20a, miR-214	Wnt/β-catenin	[67]
hsa_circ_001988	FBXW7	–	Downregulated	–	–	[68]
circ-BANP	BANP	–	Upregulated	–	p-Akt	[75]
circ-HIPK3	HIPK3	miRNA sponge	Upregulated	miR-7	FAK, IGF1R, EGFR, YY1	[76]
hsa_circ_0001649	SHPRH	–	Downregulated	–	–	[69]
hsa_circ_0007534	DDX42	–	Upregulated	–	–	[77]
hsa_circ_0003906	–	–	Downregulated	–	–	[70]
hsa_circ_000984	CDK6	miRNA sponge	Upregulated	miR-106b	CDK6	[78]
hsa_circ_0020397	DOCK1	miRNA sponge	Upregulated	miR-138	TERT, PD-L1	[79]

### HCC

Studies have shown that circRNAs are involved in the development of HCC, although the mechanism remains unclear. Shang et al. found 61 circRNAs expressed differentially between HCC and adjacent tissues, including 26 upregulated and 35 downregulated circRNAs [82]. Fu et al. identified 527 circRNAs in HCC and reported that most of them are downregulated in HCC. The two most significantly downregulated circRNAs are hsa_circ_0004018 encoded by SMYD4 and hsa_circ_0003570 encoded by FAM53B, which are associated with the clinicopathological features of HCC [83, 84]. Hsa_circ_0001649 [85], which is derived from the SHPRH gene, and circZKSCAN1 [86], which is derived from the ZKSCAN1 gene, are also significantly downregulated in HCC tissues. Tumor number, cirrhosis, vascular invasion, microvascular infiltration (MVI), and tumor grade are the major factors associated with the circ-ZKSCAN1 expression level. Overexpression of circZKSCAN1 inhibits HCC progression both in vitro and in vivo. The circRNA encoded by the MTO1 gene is also downregulated in HCC tissues. As a sponge of miR-9, circ-MTO1 can inhibit HCC progression by upregulating the expression of p21. Decreased expression of circ-MTO1 is associated with poor outcome in HCC patients, and intratumoral administration of circ-MTO1 siRNA promotes HCC growth in vivo, indicating that circ-MTO1 is a potential prognostic marker and therapeutic target for HCC [87]. The low expression of circ-ITCH in HCC is also associated with a shorter OS [88]. Hsa_circ_0001649 was also lowly expressed in HCC tissues compared to normal tissues, and the expression level of hsa_circ_0001649 is related to tumor size and tumor thrombus. Knockdown of hsa_circ_0001649 increases the levels of MMP9, MMP10, and MMP13, suggesting that it may be a protective factor for HCC metastasis [85] and can be used as a potential diagnostic and prognostic marker. Moreover, hsa_circ_0001445 (cSMARCA5), hsa_circ_0005986, and hsa_circ_0067531 [89] are also downregulated in HCC. CSMARCA5 is regulated by DHX9, and it promotes the expression of TIMP3 and inhibits the proliferation and metastasis of HCC cells by adsorbing miR-17 and miR-181b [90]. HCC patients with cSMARCA5 low expression are usually accompanied by shorter OS and RFS. The expression level of hsa_circ_0005986 in HCC cell lines, including HepG2, Huh7, SMMC7721, HCCLM3, MHCC97H, and MHCC97L, is significantly lower than that in the L02 normal liver cell line. Both hsa_circ_0005986 and Notch1 mRNA can bind to miR-129-5p, and downregulation of hsa_circ_0005986 releases miR-129-5p to decrease the level of Notch1 mRNA, accelerating the cell proliferation by promoting G0/G1 to S phase transition [91].

By using a circRNA chip, Huang et al. identified 189 circRNAs significantly upregulated and 37 circRNAs downregulated in HCC compared to adjacent tissues. circRNA_100338 acts as an endogenous sponge of miR-141-3p in HCC to regulate the invasion function of hepatoma cells, and its high expression is closely related to a poorer OS and PFS of HCC patients [92]. ciRS-7 (hsa_circ_0001946) is significantly upregulated in HCC tissues [93] and is negatively correlated with miR-7 expression [94]. Overexpression of ciRS-7 is a risk factor for MVI in the liver. When ciRS-7 is knocked down, miR-7 is released and proliferation and invasion of HCC cells are also significantly inhibited [93, 94]. However, Xue et al. found that ciRS-7 is downregulated in HCC cells and tissues [95]. Zheng et al. reported that circRNA encoded by exon 2 of HIPK3 is upregulated in HCC tissues. circ-HIPK3 is a highly stable circRNA which can adsorb and inactivate a variety of miRNAs, including miR-124, the well-known tumor suppressor [4]. Therefore, targeting circ-HIPK3 may inhibit the growth of HCC cells in patients. Hsa_circ_0067934, which is highly expressed in HCC tissues and cells, can directly adsorb miR-1324, affect the expression level of FZD5, and regulate the Wnt/β-catenin signaling pathway. Knockdown of has_circ_0067934 significantly inhibits the proliferation, invasion, and metastasis of Hep3B and HuH7 cells and induces apoptosis [96]. Hsa_circ_0005075 is considered to be closely related to cell adhesion, which is an important part of tumor cell proliferation and metastasis. A recent study showed that the expression level of hsa_circ_0005075 is significantly different between HCC and normal liver tissues and is related to HCC tumor size. Larger tumor sizes correlated with higher expression of hsa_circ_0005075. Thus, hsa_circ_0005075 has the potential to become an ideal biomarker for HCC. Hsa_circ_0005075 has binding sites for four miRNAs, namely, miR-23a-5p, miR-23b-5p, miR-93-3p, and miR-581, in which miR-23b-5p is a key factor [82]. The specific molecular mechanism of hsa_circ_0005075 as the miR-23b-5p sponge to regulate the development of HCC remains to be further studied. The combination of circRNAs and traditional biomarkers of HCC will have greater diagnostic value. The expression and function of circRNAs in HCC are shown in Table 7.

**Table 7:** circRNAs in HCC

circBase ID (alias)	Host gene	Putative function	Upregulated/downregulated	miRNA sponge	Target gene/pathway	Reference
hsa_circ_0001649	SHPRH	–	Downregulated	–	–	[85]
hsa_circ_0001727	ZKSCAN1	miRNA sponge*	Downregulated	–	–	[86]
hsa_circ_0005075	EIF4G3	miRNA sponge*	Upregulated	–	–	[82]
hsa_circ_0000284	HIPK3	miRNA sponge*	Upregulated	miR-124, miR-152	–	[4]
				miR-193a, miR-29a		
				miR-29b, miR-338		
				miR-379, miR-584		
				miR-654		
hsa_circ_0007874	MTO1	miRNA sponge	Downregulated	miR-9	p21	[87]
hsa_circ_0004018	SMYD4	miRNA sponge*	Downregulated	–	–	[83]
hsa_circ_0003570	FAM53B	–	Downregulated	–	–	[84]
hsa_circ_0001946	CDR1	miRNA sponge	Upregulated	miR-7	CCNE1, PIK3CD	[93]
hsa_circ_0001946	CDR1	miRNA sponge	Upregulated	miR-7	–	[94]
has_circ_0067934	–	miRNA sponge	Upregulated	miR-1324	FZD5/Wnt/β-catenin	[96]
hsa_circ_0067531	PIK3CB	–	Downregulated	–	–	[89]
hsa_circ_0001445	SMARCA5	miRNA sponge	Downregulated	miR-17, miR-181b	TIMP3	[90]
hsa_circ_0001946	CDR1	miRNA sponge	Downregulated	miR-7	EGFR	[95]
hsa_circRNA_100,338	–	miRNA sponge	Upregulated	miR-141	–	[92]
hsa_circ_0005986	PRDM2	miRNA sponge	Downregulated	miR-129	Notch1	[91]

*Not validated experimentally

### Brain glioma

Song et al. selected 476 differentially expressed circRNAs from the RNA-seq data of 46 cases with brain glioma. The expression levels of circ_COL1A2, circ_PTN, circ_VCAN, circ_PLOD2, circ_SMO, circ_CLIP2, circ_GLIS3, and circ_EPHB4 in glioblastoma (GBM) are significantly higher than those in normal tissues. These circRNAs may act as miRNA sponges, which in turn increase the expressions of certain genes involved in pathological processes. The circRNA derived from the VCAN gene is associated with the development of gliomas, and it is upregulated in oligodendrogliomas and GBM [97]. Barbagallo et al. [98] found that miR-671-5p is overexpressed in GBM cells and tissues, which is associated with downregulation of ciRS-7. Hsa_circ_0046701 is highly expressed in glioma tissues. Silencing hsa_circ_0046701 upregulates miR-142, resulting in a decrease of ITGB8 and inhibition of cell proliferation and invasion. The hsa_circ_0046701/miR-142/ITGB8 axis may contribute significantly to the development of gliomas [99]. circ-SHKBP1 is highly expressed in high-grade gliomas, and knockdown of circ-SHKBP1 significantly inhibits cell proliferation and metastasis. The regulation of cell function by circ-SHKBP1 is achieved by targeted uptake of miR-544a/miR-379 and upregulation of FOXP1/FOXP2 [100].

In addition, the expression levels of circZNF292 [101] and TTBK2 gene-derived circRNAs [102] in gliomas are downregulated and upregulated, respectively. Under hypoxic conditions, cZNF292 is a circRNA expressed in endothelial cells involved in glioma cell proliferation and tube formation. Silencing cZNF292 inactivates the Wnt/β-catenin signaling pathway in U87MG and U251 cells, thereby arresting cell cycle and inhibiting cell proliferation [101]. Hsa_circ_022705 (circ-FBXW7) is lowly expressed in glioma tissues and cells, and it is positively correlated with the prognosis of patients with glioma. In addition, hsa_circ_022705 encodes the FBXW7-185aa protein. Upregulation of FBXW7-185aa significantly inhibits the proliferation of tumor cells, while silencing this protein promotes the malignant phenotype [103]. circ-SMARCA5 is significantly downregulated in gliomas and negatively correlated with the histological grade of gliomas. Overexpression of circ-SMARCA5 significantly decreases the metastatic capacity of U87MG cells. circ-SMARCA5 has abundant binding motifs with several RBPs, and it can directly bind to SRSF1 and regulate its expression [104]. circ-SHPRH is abundantly expressed in normal human brain, but it is significantly downregulated in GBM. It has the ability to encode the SHRH-146aa protein with the help of an open reading frame (ORF) driven by an internal ribosome entry site (IRES). Overexpression of SHRH-146aa in U251 and U373 GBM cells reduces the malignant phenotype [105].

The expression of circ-TTBK2 is increased in glioma tissues, which promotes cell proliferation, migration, and invasion but inhibits apoptosis [102]. In addition, circ-BRAF is significantly reduced in glioma patients with a higher pathological grade. The high expression of circ-BRAF is an independent predictive marker for PFS and OS in glioma patients [106]. In the future, more research is needed to reveal the regulatory mechanism of circRNAs in gliomas. The expression and function of circRNAs in gliomas are shown in Table 8.

**Table 8:** circRNAs in glioma

circBase ID (alias)	Host gene	Putative function	Upregulated/downregulated	miRNA sponge	Target gene/pathway	Reference
circ-VCAN	VCAN	–	Upregulated	–	–	[97]
circ-ZNF292	ZNF292	–	Downregulated	–	Wnt/β-catenin	[101]
hsa_circ_0000594	TTBK2	miRNA sponge	Upregulated	miR-217	HNF1β/Derlin-1	[102]
hsa_circ_0001946	CDR1	miRNA sponge	Downregulated	miR-671	–	[98]
circ-BRAF	BRAF	–	Downregulated	–	–	[106]
hsa_circ_0046701	YES1	miRNA sponge	Upregulated	miR-142	ITGB8	[99]
circ-SHKBP1	SHKBP1	miRNA sponge	Upregulated	miR-544a, miR-379	FOXP1, FOXP2	[100]
circ-FBXW7	FBXW7	Translating protein	Downregulated	–	–	[103]
hsa_circ_0001445	SMARCA5	RNA-binding proteins	Downregulated	–	SRSF1	[104]
circ-SHPRH	SHPRH	Translating protein	Downregulated	–	–	[105]

### Bladder cancer

Bladder cancer is a common urinary system malignancy, especially in males [107]. The circRNAs in bladder cancer show an overall upregulation, indicating that there are more upregulated circRNAs than downregulated circRNAs in bladder cancer tissues [108–110]. High-throughput microarray analysis has been used to identify six circRNAs that are differentially expressed in bladder cancer and normal tissues as follows: circPTK2 (hsa_circ_0005273), circTCF25 (hsa_circ_0041103), circBC048201 (hsa_circ_0061265), and circZFR (hsa_circ_0072088) are significantly upregulated and circTRIM24 (hsa_circ_0082582) and circFAM169A (hsa_circ_0007158) are downregulated [108]. The gene expression profiles of the linear transcripts corresponding to the overexpressed circRNAs are favorable for protein modification, binding, and intracellular metabolic processes, while those downregulated circRNAs are favorable for molecular function and catalytic activity [111]. Overexpression of circTCF25 increases CDK6 expression via adsorbing miR-103a-3p and miR-107, and it promotes cell proliferation and migration [108]. Both circRNA-MYLK and vascular endothelial growth factor (VEGF) A are significantly upregulated in bladder cancer. circRNA MYLK directly binds to miR-29a and reduces its ability to target VEGFA, a molecule that activates the VEGFA/VEGFR2 signaling pathway. Functionally, overexpression of circRNA MYLK promotes cell proliferation, migration, tube formation, and rearrangement of cytoskeleton [112].

Several circRNAs are downregulated in bladder cancer. For example, circ-ITCH is downregulated in bladder cancer tissues and cell lines, and patients with low circ-ITCH expression are significantly associated with a shorter OS. Overexpression of circ-ITCH inhibits the malignant biological behavior of bladder cancer cells, such as proliferation, migration, invasion, and metastasis, by upregulating p21 and PTEN through the uptake of miR-17 and miR-224 [113]. The expression of circ-BCRC4 in bladder cancer tissues is also lower than that in adjacent normal tissues. Overexpression of circ-BCRC4 inhibits the level of miR-101, thereby upregulating EZH2 expression, which promotes the apoptosis and inhibits the activity of T24T and UMUC3 cells [114]. circ-HIPK3 is expressed at a low level in bladder cancer cells and tissues, and it is negatively associated with differentiation, infiltration, and lymph node metastasis. Overexpression of circ-HIPK3 effectively inhibits growth, migration, invasion, and angiogenesis. circ-HIPK3 contains two key binding sites for miR-558, which significantly adsorbs miR-558, thereby inhibiting HPSE expression [110]. The expression and function of circRNAs in bladder cancer are shown in Table 9.

**Table 9:** circRNAs in bladder cancer

circBase ID (alias)	Host gene	Putative function	Upregulated/downregulated	miRNA sponge	Target gene/pathway	Reference
hsa_circ_0041103	TCF25	miRNA sponge	Upregulated	miR-103a, miR-107	CDK6	[108]
hsa_circ_0002768	MYLK	miRNA sponge	Upregulated	miR-29a	VEGFA/VEGFR2	[112]
circ-ITCH	ITCH	miRNA sponge	Downregulated	miR-17, miR-224	p21, PTEN	[113]
circ-BCRC4	BCRC4	miRNA sponge	Downregulated	miR-101	EZH2	[114]
circ-HIPK3	HIPK3	miRNA sponge	Downregulated	miR-558	HPSE	[110]

### Pancreatic cancer

In pancreatic ductal adenocarcinoma (PDAC), 351 differentially expressed circRNAs (including 209 upregulated and 142 downregulated) between cancer tissues and normal tissues are identified by microarray analysis. Hsa_circ_0000977 is abnormally upregulated in pancreatic cancer tissues, and silencing hsa_circ_0000977 inhibits cell proliferation and induces cell cycle arrest. The interaction of hsa_circ_0000977, hsa-miR-874-3p, and PLK1A has been verified by dual luciferase reporter assay and fluorescence in situ hybridization (FISH) assay, and inhibition of hsa_circ_0000977 can reduce the expression of PLK1. In animal experiments, silencing hsa_circ_0000977 inhibits tumor growth [115]. circ-LDLRAD3 is overexpressed in cells, tissues, and plasma samples of pancreatic cancer patients. High expression of circ-LDLRAD3 is significantly correlated with venous or lymphatic infiltration and metastasis, and it may be used as a diagnostic biomarker for pancreatic cancer [116]. Hsa_circ_0000284 (circRNA_100782) is significantly upregulated in PDAC tissues, which is a sponge of miR-124. Knockdown of circRNA_100782 inhibits the proliferation and colony formation of BxPC3 cells by downregulating the target genes of miR-124, namely, IL6R and STAT3 [117]. In addition, hsa_circ_0005785 has binding sites for miR-181a and miR-181b [118]. Since miR-181a and miR-181b are associated with the growth/migration and gemcitabine resistance of pancreatic cancer cells, respectively, hsa_circ_0005785 may be involved in PDAC progression and gemcitabine resistance. The expression and function of circRNAs in pancreatic cancer are shown in Table 10.

**Table 10:** circRNAs in pancreatic cancer

circBase ID (alias)	Host gene	Putative function	Upregulated/downregulated	miRNA sponge	Target gene/pathway	Reference
hsa_circ_0005397	RHOT1	miRNA sponge*	Upregulated	miR-26b, miR-125a, miR-181a, miR-330, miR-382	–	[118]
hsa_circ_0005785	ANAPC7	miRNA sponge*	Downregulated	miR-181a/b/d, miR-338, miR-526b	–	[118]
hsa_circ_0000977	NOL10	miRNA sponge*	Upregulated	miR-874	PLK1	[115]
circ-LDLRAD3	LDLRAD3	–	Upregulated	–	–	[116]
hsa_circ_0000284	HIPK3	miRNA sponge	Upregulated	miR-124	IL6/STAT	[117]

*Not validated experimentally

### Osteosarcoma

The circRNA encoded by oncogenic KCNH1 is upregulated in osteosarcoma tissues and cells, and it promotes cell proliferation, invasion, and metastasis [119]. The circRNA encoded by UBAP2 is the most prominently upregulated circRNA in osteosarcoma tissues, and patients with high circUBAP2 expression are often associated with a poor OS. In vitro and in vivo experiments have shown that circ-UBAP2 promotes osteosarcoma cell growth and inhibits apoptosis [120]. Hsa_circ_0001564 is significantly overexpressed in osteosarcoma tissues and cells. Knockdown of hsa_circ_001564 significantly inhibits osteosarcoma cell proliferation by inducing G0/G1 cell cycle arrest and promotes apoptosis of HOS and MG-63 cells. Hsa_circ_0001564 mediates tumorigenesis as a sponge of miR-29c-3p, and miR-29c can reverse the oncogenic effects of hsa_circ_001564 [121]. Hsa_circ_0009910 is also overexpressed in osteosarcoma cells. Knockdown of circ_0009910 inhibits the proliferation of osteosarcoma cells, leading to cell cycle arrest and apoptosis. However, inhibition of miR-449a eliminates this effect. As the sponge of miR-449a, circ_0009910 upregulates the functional target gene IL6R and promotes the development of osteosarcoma [122]. In osteosarcoma cells and tissues, hsa_circRNA_103801 is upregulated, while hsa_circRNA_104980 is downregulated. The potential target miRNAs for hsa_circRNA_103801 include hsa-miR-338-3p, hsa-miR-370-3p, and hsa-miR-877-3p, which are involved in the HIF-1, Rap1, PI3K-Akt, VEGF, and angiogenesis pathways. The potential target miRNAs for hsa_circRNA_104980 are hsa-miR-660-3p and hsa-miR-1298-3p, which participate in the tight junction pathway [123]. The expression and function of circRNAs in osteosarcoma are shown in Table 11.

**Table 11:** circRNAs in osteosarcoma

circBase ID (alias)	Host gene	Putative function	Upregulated/downregulated	miRNA sponge	Target gene/pathway	Reference
hsa_circ_0016347	KCNH1	miRNA sponge	Upregulated	–	–	[119]
circ-UBAP2	UBAP2	miRNA sponge	Upregulated	miR-143	–	[120]
hsa_circ_0001564	CANX	miRNA sponge	Upregulated	miR-29c	–	[121]
hsa_circ_0009910	MFN2	miRNA sponge	Upregulated	miR-449a	JAK1/STAT3	[122]
hsa_circRNA_103801	–	miRNA sponge*	Upregulated	miR-370	–	[123]
hsa_circRNA_104980	–	miRNA sponge*	Downregulated	–	–	[123]

*Not validated experimentally

### Other tumors

The expression and function of circRNAs in other tumors are shown in Table 12.

**Table 12:** circRNAs in other tumors

circBase ID (alias)	Host gene	Putative function	Type of cancer	Upregulated/downregulated	miRNA sponge	Target gene/pathway	Reference
hsa_circ_0075828	LINC00340	miRNA sponge	BCC	Upregulated	–	–	[127]
hsa_circ_0075825	LINC00340	miRNA sponge	BCC	Upregulated	–	–	[127]
hsa_circ_0022383	FADS2	miRNA sponge	BCC	Downregulated	–	–	[127]
			CSCC	Downregulated	–	–	[128]
hsa_circ_0022392	FADS2	miRNA sponge	BCC	Downregulated	–	–	[127]
			CSCC	Downregulated	–	–	[128]
hsa_circ_0070933	LARP1B	miRNA sponge	CSCC	Upregulated	–	–	[128]
hsa_circ_0070934	LARP1B	miRNA sponge	CSCC	Upregulated	–	–	[128]
circ-DOCK1	DOCK1	miRNA sponge	OSCC	Upregulated	miR-196a	BIRC3	[130]
hsa_circ_0013339	SLC30A7	miRNA sponge	OSCC	Upregulated	miR-29b	CDK6	[129]
hsa_circ_0001649	SHPRH	miRNA sponge	Cholangiocarcinoma	Downregulated	–	MMP9	[134]
Cdr1as	CDR1	–	Cholangiocarcinoma	Upregulated	–	–	[133]
hsa_circ_0000284	HIPK3	miRNA sponge	Cervical cancer	Upregulated	miR-506	Snail-2	[135]
circ-SMARCA5	SMARCA5	–	Prostate cancer	Upregulated	–	–	[136]

#### Ovarian cancer

Ahmed et al. identified a total of 67,580 candidate circRNAs from primary and metastatic lesions of three patients with stage IIIC ovarian cancer, and they confirmed that the differential expression of circRNAs between primary and metastatic ovarian cancer is more pronounced than corresponding mRNAs [124]. Bachmayr-Heyda et al. found that the levels of circRNAs in immortalized normal ovarian epithelial cells are generally lower than those of ovarian cancer cells [65] because immortalized normal ovarian epidermal cells proliferate faster than ovarian cancer cells, resulting in reduced accumulation of circRNAs. In addition, the levels of circRNAs in miliary tumors are lower than those of non-miliary tumors; however, it cannot be explained by the difference in proliferation rates.

#### Kidney cancer

In clear cell renal cell carcinoma (ccRCC) tissues, the circRNA (circ-HIAT1) derived from hippocampus-rich gene transcription protein 1 (HIAT1) is downregulated. Compared with metastatic ccRCC, circ-HIAT1 is expressed at a higher level in non-metastatic ccRCC. In addition, the OS rate of ccRCC patients with high expression of circ-RIAT1 is superior to that of patients with low circ-RIAT1. circ-HIAT1 can directly bind to miR-29a-3p, miR-29c-3p, and miR-195-5p and upregulate the expression of CDC42. Different from the classical function of miRNA sponges, circ-HIAT1 acts as a “miRNA reservoir,” which increases miRNA stability, thereby reversing androgen receptor (AR)-mediated ccRCC migration and invasion. Inhibition of the miR-29a-3p/29c-3p/195-5p signaling pathway by circ-HIAT1 inhibits the migration and invasion of ccRCC cells [125]. The AR/circHIAT1/CDC42 signaling pathway may become a new therapeutic target for metastatic ccRCC.

#### Thyroid cancer

Compared with normal thyroid tissues, researchers have found 88 significantly upregulated circRNAs and 10 downregulated circRNAs in papillary thyroid cancer (PTC) tissues. Based on the miRNA response elements (MREs) of these dysregulated circRNAs, a network of circRNA-miRNA interactions has been constructed by Cytoscape. The downregulated circRNA, hsa_circRNA_100395, has potential for interaction with two cancer-associated miRNAs, namely miR-141-3p and miR-200a-3p, suggesting that hsa_circRNA_100395-miR-141-3p/miR-200a-3p may be involved in the pathogenesis of PTC [126]. However, this hypothesis needs further verification.

#### BCC

Sand et al. identified 23 upregulated circRNAs and 48 downregulated circRNAs in BCC tissues [127]. Hsa_circ_0022383 and hsa_circ_0022392 are the most significantly downregulated circRNAs and derived from FADS2 gene. Similarly, the two most significantly upregulated circRNAs are also encoded by the same host gene, namely, LINC00340.

#### CSCC

Sand et al. [128] identified 143 upregulated circRNAs and 179 downregulated circRNAs in CSCC. The two most significantly downregulated circRNAs (also most significantly downregulated in BCC) are from FADS2 gene, and the two most significantly upregulated circRNAs are encoded by LARP1B gene.

#### OSCC

Numerous circRNAs are differentially expressed in OSCC tissues and adjacent tissues. At present, 280 circRNAs have been identified with more than a twofold difference in OSCC, including 139 upregulated and 141 downregulated circRNAs. Among these circRNAs, hsa_circ_0013339 (circRNA_100290) is derived from the SLC30A7 gene, and it is upregulated by approximately sevenfold in OSCC compared to normal tissues. circRNA_100290 is a sponge of the miR-29 family. Functional analysis has revealed that knockdown of circRNA_100290 reduces CDK6 expression, induces G1/S arrest, and significantly inhibits the proliferation of SCC9 cell lines. In a nude mouse model, interference with circRNA_100290 also reduces tumor growth [129]. Moreover, circ-DOCK1 regulates BIRC3 expression through competitively binding to miR-196a as a ceRNA, and it is involved in the apoptosis of OSCC cells [130].

#### LSCC

In 698 dysregulated circRNAs in LSCC (302 upregulated cases and 396 downregulated cases), hsa_circ_100855 and hsa_circ_104912 are the two most significantly up- and downregulated circRNAs, respectively. High expression of hsa_circ_100855 and low expression of hsa_circ_104912 are associated with T3–4 stage, cervical lymph node metastasis, and later clinical stage of LSCC [131]. Researchers believe that the above circRNAs are involved in the initiation and development of LSCC and that they may be helpful for the diagnosis and prognosis in clinical practice.

#### Hypopharyngeal squamous cell carcinoma (HSCC)

Cao et al. showed that 2392 circRNAs are differentially expressed between HSCC and normal tissues [132]. Of these circRNAs, 1304 are upregulated, including hsa_circ_0024108, hsa_circ_0058106, and hsa_circ_0058107, while 1088 are downregulated, including hsa_circ_0001189, hsa_circ_0002260, and hsa_circ_0036722. However, the functions of these circRNAs in HSCC remain unexplored.

#### Cholangiocarcinoma

The expression of ciRS-7 (Cdr1as) in cholangiocarcinoma tissues is higher than that in adjacent normal tissues. Overexpression of ciRS-7 is closely related to later TNM staging, lymph node infiltration, and postoperative recurrence. The OS of cholangiocarcinoma patients with high ciRS-7 expression is inferior to that of patients with low ciRS-7 expression. Based on multivariate analysis, ciRS-7 is an independent prognostic biomarker with excellent sensitivity and specificity for cholangiocarcinoma [133]. Hsa_circ_0001649 is abnormally down-expressed in cholangiocarcinoma cells and tissues, and it is related to tumor size and differentiation grade of cholangiocarcinoma. Overexpression of hsa_circ_0001649 inhibits cell proliferation, migration, and invasion but induces apoptosis of KMBC and Huh-28 cells. Silencing hsa_circ_0001649 leads to the opposite effect. Therefore, hsa_circ_0001649 may be a potential diagnostic and therapeutic target for cholangiocarcinoma [134].

#### Cervical cancer

circRNA-000284 is significantly upregulated in cervical cancer cells. It promotes the proliferation and invasion of cervical cancer cells and that knockdown of circRNA-000284 causes G0/G1 cell cycle arrest, resulting in inhibition of cell proliferation and invasion. miR-506 is a miRNA related to circRNA-000284, and circRNA-000284 positively regulates the expression of Snail-2 which is a target gene of miR-506. However, co-expression of a miR-506 mimic or Snail-2 silencing vector eliminates the oncogenic effect of circRNA-000284. Thus, circRNA-000284 is expected to be a new therapeutic target for cervical cancer [135].

#### Prostate cancer

The role of circRNAs in prostate cancer is rarely explored. circ-SMARCA5 is significantly upregulated in prostate cancer tissues, and it promotes cell cycle process and inhibits apoptosis [136], acting as an oncogene.

## Discussion

Recently, the clinical significance of circRNAs in a variety of tumors has been explored. circRNAs are generally superior to the corresponding linear RNAs in terms of stability. In addition, they represent the characteristics at different stages of tumor development [83, 84, 86, 91]. In addition, circRNAs can compensate for the defect of low organ specificity of traditional biomarkers. circ-CER may serve as a diagnostic marker for NSCLC, and its overexpression is significantly associated with local lymph node invasion, advanced tumor, and poor survival [20]. The combination of hsa_circ_006054, hsa_circ_100219, and hsa_circ_406697 is helpful for the diagnosis of breast cancer [30]. Hsa_circ_0067934, a potential prognostic marker for ESCC, is overexpressed in ESCC tissues and correlates with poor differentiation and more advanced TNM staging. The upregulation of circ-PVT1 and downregulation of circ-LARP4 in gastric cancer are independent prognostic factors [45, 53], and circ-PVT1 overexpression predicts better OS and DFS. The downregulation of circMTO1, circ-ITCH, and cSMARCA5 [87, 88, 90] or upregulation of circRNA_100338 in HCC [92] is associated with poor prognosis. Overexpression of ciRS-7 in HCC tissue is a risk factor for MVI [94], and the expression of hsa_circ_0005075 in HCC is positively correlated with tumor size, suggesting that it may be a potential biomarker of HCC. Overexpression of ciRS-7 in cholangiocarcinoma is significantly correlated with later TNM staging, lymph node infiltration, and postoperative recurrence, and it may be an independent negative prognostic biomarker with good sensitivity and specificity [133]. Hsa_circ_0001649 has been reported to have potential diagnostic and prognostic value in gastric cancer, CRC, HCC, and cholangiocarcinoma [57, 69, 85, 134], and it may be a sensitive indicator for distant metastasis in gastric cancer and HCC. High expression level of circ-BRAF is an independent marker for good prognosis in glioma patients [106]. Overexpression of circ-LDLRAD3 in pancreatic cancer is significantly correlated with venous and lymphatic infiltration as well as distant metastasis, and it is also a potential diagnostic marker for pancreatic cancer [116].

Another advantage of circRNAs is that they can be easily and reproducibly detected in human blood, saliva, and gastric juices, thus increasing its potential as a biomarker [48, 137–139]. In general, many circRNAs are expressed much higher in blood than the corresponding linear RNAs. Therefore, plasma circRNAs may provide additional information that cannot be revealed by routine RNA detection. For example, hsa_circ_002059, hsa_circ_0001017, and hsa_circ_0061276 can be stably detected in the plasma of gastric cancer patients [54]. These circRNAs are expected to become convenient diagnostic biomarkers for gastric cancer. Hsa_circ_0000190 is expressed at a low level both in gastric cancer plasma and tissues, and it is related to tumor size, lymph node and distant metastasis and TNM staging. The sensitivity and specificity of hsa_circ_0000190 as a diagnostic marker for gastric cancer are even better than that of CEA and CA19-9 [55]. The plasma level of hsa_circ_0000745 in gastric cancer patients is related to lymph node metastasis, and it has a good diagnostic value in combination with CEA [63]. The contents of circRNAs in exosomes are enriched more than twofold compared to their intracellular levels [140]. Bahn et al. found 422 circRNAs involved in intercellular signaling and inflammatory responses by bioinformatics analysis in human cell-free saliva [138]. Shao et al. demonstrated that hsa_circ_0014717 is stably detected in gastric juice, not affected by freeze-thaw for eight cycles or storage at 4 °C for 8 h [48]. The expression patterns and characteristics of circRNAs give them the potential to serve as a good biomarker in a variety of tumors.

The use of circRNAs as a therapeutic target or therapeutic vector will be a future trend. In therapeutic strategies targeting oncogenic circRNAs, exogenous siRNAs that are fully complementary to the back-splice junction can be used. Alternatively, it is possible to interfere with back-splicing by antisense oligonucleotides that are complementary to the back-splice signals in the precursor mRNA. It is necessary to avoid interfering with the expression of homologous linear mRNA. Another strategy is to induce tumor suppressor circRNA expression through gene therapy. In addition, some prefabricated circRNAs independent of nuclear splicing and output may also be used for the treatment of tumors. circRNAs have extremely high stability and the ability to adsorb miRNAs and proteins, suggesting that they can serve as delivery vehicles for certain treatments. circRNAs containing binding sites with oncogenic miRNAs and/or proteins can control the proliferation of tumor cells or induce apoptosis. Some strategies may help to achieve more precise treatment, for example, restricting the expression of circRNAs to certain types of cells by cell-specific promoters or designing different combinations of circRNAs and miRNAs and/or protein binding sites according to sponge maps to target specific carcinogenic factors. Because some circRNAs serve as a template for protein expression [8, 9, 141], cassettes containing tumor suppressor proteins can convert circRNAs into an effective treatment method for tumors.

At present, the exploration of the correlation between circRNAs and various diseases, including tumors, has become a new research field. Various methods have been developed for detecting circRNAs expression and biological functions. RNA-seq and microarrays are used to determine target circRNAs. The expression of circRNAs is mainly verified by real-time quantitative polymerase chain reaction (RT-qPCR), micro-drop digital PCR, Northern blotting, and FISH. For functional studies, overexpression and knockdown of genes are commonly used to regulate the expression of circRNAs. Bioinformatics predictions, luciferase reporter assays, RNA immunoprecipitation, and RNA pull-down experiments combined with mass spectrometry are utilized to reveal the interactions of circRNA-miRNA and circRNA-protein. M6A, IRES, and ORF in circRNAs can be predicted by bioinformatics analysis to investigate the protein-encoding ability of circRNAs. Ribosomal imprinting, ribosome IP, m6A IP, Western blotting, and mass spectrometry are commonly used in validation studies [142]. The development of high-throughput RNA-seq technology and bioinformatics methods enables accurate identification and quantification of circRNAs. Some online databases, such as circBase, CircInteractome, CircNet, and Circ2Traits, provide the basic information and potential regulatory networks of circRNAs. Continuous improvement of statistics and calculation methods will aid in a clearer and more comprehensive understanding of the expression patterns of circRNAs.

## Conclusions

The present review introduced the biogenesis, characteristics, functions, and clinical value of circRNAs. circRNAs are closely related to a variety of physiological conditions and involved in certain diseases with a high degree of tissue and cell specificity. The biogenesis of circRNAs is a strictly controlled biological process, rather than a random splicing error. Although scientists have initially proposed a synthetic model for circRNAs, more research is needed to fully explore the mechanisms of circRNA generation, including the secondary structure of circRNAs, the initiation of novel circRNAs, the relationship among homologous RNA isomers, and the crosstalk between circRNAs and other molecules.

circRNAs have an extremely wide range of biological functions. As a miRNA sponge, circRNA makes the ceRNA network more complete and complicated. However, ceRNA does not represent all the functions of circRNAs. In the future, it is necessary to explore other mechanisms of circRNAs in tumors, such as regulating gene or protein activity.

The role of circRNAs in tumorigenesis and development has become the focus of oncology. circRNAs are considered as new diagnostic and prognostic biomarkers and potential therapeutic targets in the future. In the previous studies, the detection of circRNAs was mainly in tissues, but it is possible to detect the expressions of circRNAs in more accessible and less invasive samples (such as blood, urine, and saliva) or samples closely related to diseases (such as gastric juice, synovial effusion, and cerebrospinal fluid). It is necessary to develop circRNA as a clinical diagnostic biomarker based on the optimization of consistency and standardization of sample processing and detection. The combined detection of different circRNAs and traditional markers may have higher diagnostic efficiency. In addition, the potential of circRNAs in cancer therapy cannot be ignored. circRNA-targeted treatment may become a new mode of tumor therapy in the future.

Although increasingly more circRNAs have been discovered and investigated, the functions of thousands of circRNAs remain unclear. Research on circRNAs is still in its beginning stage, and only a small part of circRNA mechanism in tumorigenesis and progression has been clarified. With the efforts of scientists and application of new methods, more circRNAs will be discovered and applied in the diagnosis and treatment of related diseases, including tumors.

## Abbreviation

AR: Androgen receptor
BCC: Basal cell carcinoma
BCSCs: Breast cancer stem cells
ccRCC: Clear cell renal cell carcinoma
ceRNA: Competing endogenous RNA
circRNA: Circular RNA
CIRI: circRNA identifier
ciRNAs: Circular intronic RNAs
CRC: Colorectal carcinoma
CSCC: Cutaneous squamous cell carcinoma
DCIS: Ductal carcinomas in situ
DFS: Disease-free survival
ecircRNA: Exonic circRNA
EIciRNA: Exon-intron circRNA
ER: Estrogen receptor
ESCC: Esophageal squamous cell carcinoma
FISH: Fluorescence in situ hybridization
GBM: Glioblastoma
HCC: Hepatocellular carcinoma
HIAT1: Hippocampus-rich gene transcription protein 1
HSCC: Hypopharyngeal squamous cell carcinoma
ICFs: Intronic circRNA fragments
IDC: Invasive ductal carcinoma
IRES: Internal ribosome entry site
LSCC: Laryngeal squamous cell cancer
miRNA: MicroRNA
MPP: Matrix metalloproteinase
MREs: miRNA response elements
MVI: Microvascular infiltration
ncRNAs: Non-coding RNAs
NSCLC: Non-small cell lung cancer
ORF: Open reading frame
OS: Overall survival
OSCC: Oral squamous cell carcinoma
PDAC: Pancreatic ductal adenocarcinoma
pre-mRNA: Precursor mRNA
PTC: Papillary thyroid cancer
RBPs: RNA-binding proteins
RNA-seq: RNA sequencing
RT-qPCR: Real-time quantitative polymerase chain reaction
SA: Splice acceptor
SD: Splice donor
TN: Triple negative
VEGF: Vascular endothelial growth factor
